# STATE-LEVEL DESEGREGATION EFFORTS IN THE U.S. SOUTH POST-BROWN AND DEMENTIA RISK AMONG BLACK AND WHITE OLDER ADULTS

**DOI:** 10.1093/geroni/igac059.204

**Published:** 2022-12-20

**Authors:** Katrina Walsemann, Mateo Farina, Jennifer Ailshire

**Affiliations:** University of Maryland, College Park, Maryland, United States; University of Southern California, Los Angeles, California, United States; University of Southern California, Los Angeles, California, United States

## Abstract

Although education is a key protective factor against dementia, older U.S. adults experienced vastly different educational contexts. One of the most consequential legal decisions impacting educational contexts was Brown v. Board of Education, which declared ‘separate but equal’ schools unconstitutional. School desegregation in the U.S. South was inconsistent, however, and many states delayed implementation for years. State resistance to school desegregation created school environments for Black children that were stressful and discriminatory. Thus, state variation in exposure to school desegregation may serve to differentiate individuals in their risk for dementia. Our project links historical data on state-level school desegregation efforts to the Health and Retirement Study, a nationally representative sample of U.S. adults over 50. We determine if state-level variation in the timing and completeness of school desegregation explains Black-White disparities in transition to cognitive impairment and dementia among older adults who attended school in the US South post-Brown.

